# Experimental evidence for enzymatic cell wall dissolution in a microbial protoplast feeder (*Orciraptor agilis*, Viridiraptoridae)

**DOI:** 10.1186/s12915-022-01478-x

**Published:** 2022-12-05

**Authors:** Jannika Moye, Tobias Schenk, Sebastian Hess

**Affiliations:** Institute for Zoology, University of Cologne, Zülpicher Str. 47b, 50674 Cologne, Germany

**Keywords:** Cell wall, Cellulase, Phagocytosis, Protist, Protozoa, Rhizaria, Zygnematophyceae

## Abstract

**Background:**

Several protists have evolved the ability to perforate the cell walls of algae and fungi to specifically feed on their cell contents. These phagotrophic “protoplast feeders” represent an interesting mechanistic intermediate between predators and parasites and pose a number of cell biological questions. Although their fascinating feeding behaviour has been observed for the last 150 years, it is still unknown how protoplast feeders produce the well-defined and species-specific perforations in biochemically diverse cell walls. Differential expression analyses of the algivorous flagellate *Orciraptor agilis* (Viridiraptoridae, Cercozoa, Rhizaria) suggested the involvement of a highly expressed putative glycoside hydrolase of family GH5_5. To assess the importance of this carbohydrate-active enzyme in the feeding act of *Orciraptor*, we recombinantly produced its catalytic domain and studied the enzymatic activity, cellular localisation and function.

**Results:**

The GH5_5 catalytic domain from *Orciraptor* showed pronounced activity on soluble cellulose derivatives and mixed-linkage glucans, with reaction optima comparable to known GH5_5 representatives. Crystalline cellulose was not digested by the enzyme, which suggests a typical endocellulase activity. Immunocytochemistry with a polyclonal antibody raised against the GH5_5 domain revealed that the native endocellulase localises to the contact zone of *Orciraptor* and the algal cell wall (= perforation zone) and to intracellular granules, which were enriched during attack. Furthermore, the anti-GH5_5 antibody applied to live cells significantly reduced the feeding success of *Orciraptor*. The cells attacked the algae, which, however, resulted in numerous incomplete perforations.

**Conclusions:**

Our experimental data from enzymatic assays, immunocytochemistry and inhibition experiments strongly suggest a key role of the GH5_5 endocellulase in cell wall dissolution by *Orciraptor agilis*. With that, we provide evidence that the well-defined perforations produced by protoplast feeders are caused by extracellular carbohydrate-active enzymes and made a first step towards establishing the molecular basis of a fascinating, yet poorly understood microbial feeding strategy.

**Supplementary Information:**

The online version contains supplementary material available at 10.1186/s12915-022-01478-x.

## Background

Microbes have evolved various strategies to capitalise on other organisms. Some phagotrophic protists, referred to as “protoplast feeders”, specifically feed on the cell contents of other eukaryotes by penetrating the foreign cell wall. They are phylogenetically diverse and can be found in several eukaryotic supergroups; for example, in the Rhizaria (e.g. vampyrellid amoebae, viridiraptorid flagellates), Amoebozoa (e.g. *Idionectes vortex*, Schizoplasmodiidae), and Opisthokonta (e.g. Aphelidea, Nucleariidae) [1–6]. The highly specialised feeding processes of protoplast feeders typically involves (1) prey cell recognition, (2) attachment, (3) local dissolution of the cell wall and (4) phagocytosis of the cell contents. Some species extract the cell contents of their prey by pseudopodial movements [7–13], while others infiltrate, feed and complete their life history inside the prey cell [1, 2, 4, 14]. In most known cases, the contents of the prey cell (e.g. vacuoles, plastids) disintegrate during the attack by a protoplast feeder, which is very obvious in freshwater green algae. After the local weakening of the wall by the protoplast feeder, the internal pressure (turgor) of the algal cell causes the cell to burst, i.e. the rapid ejection of the cell contents [10]. Some vampyrellid amoebae, in particular, are able to simultaneously take up the ejected material in a large food vacuole, which resembles a sucking motion and might be responsible for their figurative name [10, 15]. Other protoplast feeders, e.g. the cercozoan flagellate *Orciraptor agilis*, specifically feed on the contents of dead algal cells, which usually do not burst upon attack [2]. Hence, protoplast feeders can have various ecological roles, for example, as predators (extracting unicellular prey), parasitoids (infiltrating cells of multicellular organisms), and necrophages (consuming the contents of dead cells). However, they all rely on the local, pre-phagocytotic dissolution of a foreign cell wall.

Protoplast feeders target prey of diverse phylogenetic affinity and cell wall biochemistry. The prey organisms include chlorophyte and streptophyte green algae with cellulose- and pectin-rich walls [15–18], chlorodendralean green algae with thecae composed of unusual keto-sugar acids [7], fungal hyphae and spores with beta-glucans, chitin and melanin [9, 19, 20] and diatoms with siliceous frustules [8]. Except for diatom frustules, protoplast feeders produce well-defined perforations of species-specific size and outline. A main distinguishing feature of these perforations is the pattern of cell wall dissolution, which eventually leads to the opening of sufficient size for the feeding act. “Type I” perforations are based on a uniform cell wall dissolution in a circular or elliptical area, while “type II” perforations result from an annular degradation zone and involve the formation of an “excised” cell wall disc (sometimes referred to as “lid” or “operculum”) [3]. The annular cell wall degradation might be an efficient way of creating large holes with a minimum investment of cellular resources and time, and it has been documented in several distinct groups of protoplast feeders that feed on algae and fungi [2, 19–21].

Protoplast feeding has been known for more than 150 years [15], but the molecular processes underpinning this fascinating microbial behaviour are still unknown. It differs fundamentally from other microbial interactions such as those known from parasitic fungi, oomycetes and plasmodiophorids [22–24]. Based on light and electron microscopic observations on vampyrellid amoebae (Vampyrellida, Rhizaria), some authors suggested the action of lytic enzymes [9, 12, 25, 26], but there is no experimental evidence. More recently, comparative transcriptomics applied to the necrophagous protoplast feeder *Orciraptor agilis* (Viridiraptoridae, Rhizaria) revealed a hypothetical protein with a domain annotated as glycoside hydrolase of family GH5_5 [27]. This candidate protein (referred to as GH5_5A) was the highest expressed carbohydrate-active enzyme (CAZyme) of *Orciraptor*, strongly upregulated during attack on the green alga *Mougeotia* sp. (Zygnematophyceae), and hence suspected to be a key factor for cell wall dissolution. The degradation of the plant-like, cellulosic walls of zygnematophytes likely requires glycoside hydrolases that cleave β-1,4-glycosidic bonds, a function that was predicted for *Orciraptor’s* GH5_5A protein. The open reading frame (ORF) of GH5_5A is relatively long (2248 amino acids) and further contains an N-terminal signal peptide, a C-terminal transmembrane domain, and a series of seven related sequence motifs (repeats), some of which were weakly annotated as cellulose-binding domain CBM2 [27]. This points to a dual function (catalytic and binding) of GH5_5A and suggests an extracellular localisation, which would be required for dissolving the algal cell wall.

Here, we corroborated the gene structure of GH5_5A by PCR (the ORF was not fully assembled in the first place, [27]) and studied the activity, substrate specificity and cellular localisation of the GH5_5 catalytic domain. For this, we established an optimised experimental system with *Orciraptor agilis* and the unicellular desmid *Actinotaenium* cf. *silvae-nigrae* (Zygnematophyceae), produced the GH5_5 domain recombinantly in prokaryotic and eukaryotic hosts and generated a custom polyclonal antibody (anti-GH5_5 pAb) for immunolocalisation. Our results on the activity and cellular localisation of GH5_5A, and pronounced effects of the anti-GH5_5 pAb on *Orciraptor’s* feeding process, provide unprecedented insights into the biochemical basis of cell wall dissolution in protoplast feeders.

## Results

### Feeding of *Orciraptor agilis* on *Actinotaenium* cf. *silvae-nigrae*

**Fig. 1:**
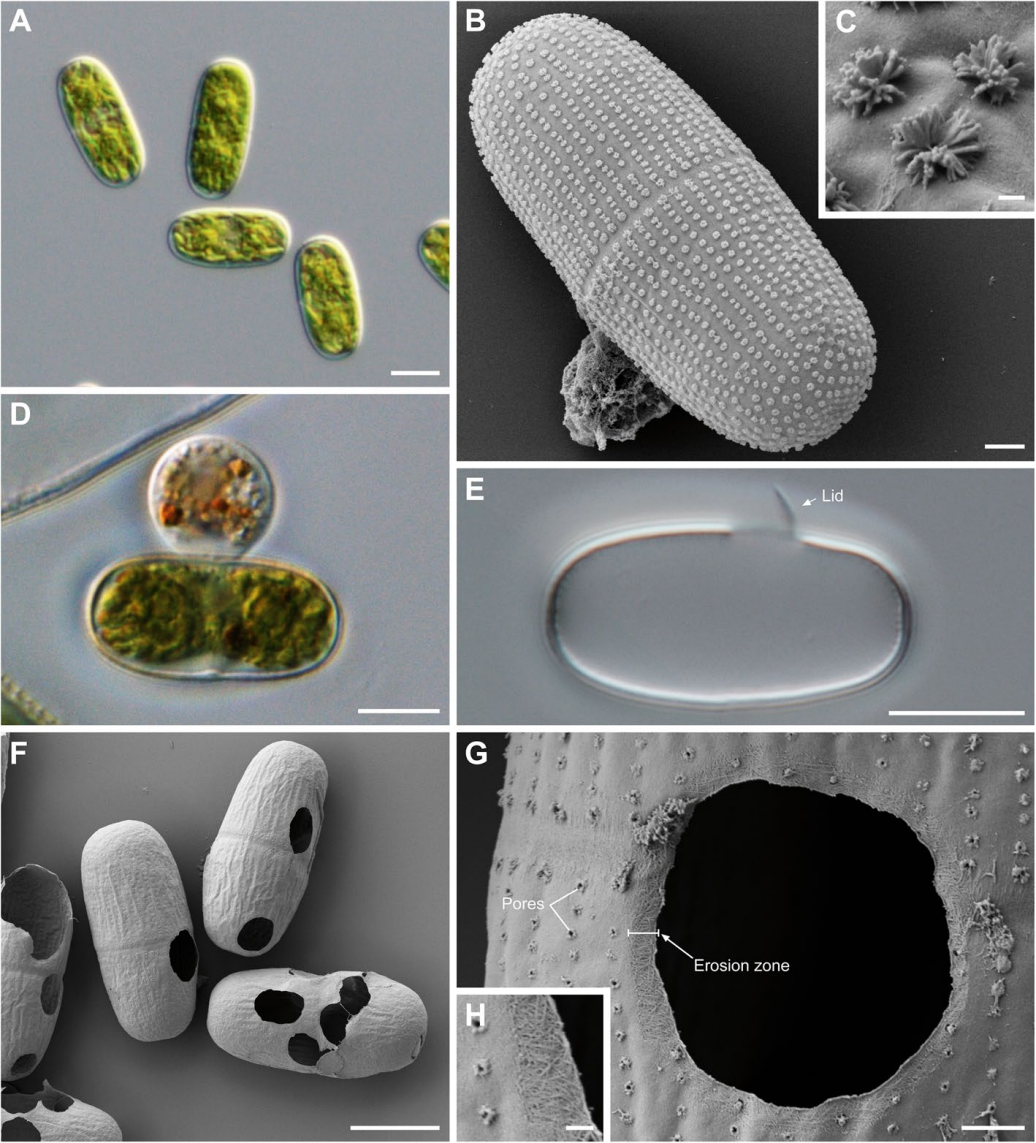
Structure of *Actinotaenium* cf. *silvae-nigrae* (strain CCAC 0140) and cell wall perforations produced by *Orciraptor agilis*. **A** Morphology of live *Actinotaenium* cells, DIC. **B** Surface of intact *Actinotaenium* cell with longitudinal rows of rosette-like ornamentations, SEM. **C** Detail of rosettes on the surface of *Actinotaenium*, SEM. **D ***Orciraptor* attacking a dead *Actinotaenium* cell, DIC. **E** Empty *Actinotaenium* cell with perforation and attached cell wall disc excised by *Orciraptor*, DIC. **F** Empty and perforated *Actinotaenium* cells that remained in an *Orciraptor* culture, SEM. **G** Detail of a perforation produced by *Orciraptor* with degraded rosettes (revealing cell wall pores) and eroded cellulose microfibrils, SEM. **H** Magnified erosion zone of G. Scale bars in **A**, **D**, **E**, **F** = 10 μm, in **B** = 2 μm, in **C**, **H** = 200 nm, in **G** = 1 μm

*Orciraptor agilis* feeds on a range of algal species [2]. To optimise our experimental system, we studied the interaction of *Orciraptor* with the unicellular desmid *Actinotaenium* cf. *silvae-nigrae*, strain CCAC 0140 (Fig. 1A). In contrast to the filamentous alga *Mougeotia* sp., which was used in previous experiments [27, 28], the *Actinotaenium* cells have a relatively high sinking rate and can be easily counted. Furthermore, the cell wall of *Actinotaenium* cf. *silvae-nigrae* is decorated with distinctive rosette-like structures (“rosettes” in the following), which help to assess alterations of the cell wall (Fig. 1B, C). These rosettes were always arranged in longitudinal rows and varied in size between algal cells, potentially relating to the age of the (half-)cells of the desmids. The chemical composition of the rosettes is unknown. *Orciraptor* readily attacked dead *Actinotaenium* cells and formed its characteristic lysopodium [28] (Fig. 1D). The perforation process took at least 4 h (in contrast to 45 min with *Mougeotia* sp.), probably owing to the relatively thick cell wall of *Actinotaenium*. As observed in other algae [2, 28], the perforation pattern was ring-like and resulted in an “excised” cell wall disc or a lid-like structure remaining at the emptied *Actinotaenium* cells (Fig. 1E). Scanning electron microscopy (SEM) revealed that all three structural components of the algal cell wall, i.e. rosettes, smooth pectin-like substances and cellulose microfibrils, were degraded after contact with *Orciraptor* (Fig. 1F, G). The size of the rosettes decreased with proximity to the perforation zone, indicating varying degradation, and sometimes the underlying cell wall pores became visible (Fig. 1G). In most cells, the exposed and degraded cellulose microfibrils were confined to a relatively narrow zone of < 0.5 μm at the inner margin of the cell wall holes (Fig. 1G, H). The surface of the excised cell wall discs and lids showed varying degradation, ranging from eroded rosettes to exposed cellulose microfibrils (Additional file 1: Fig. S1).

### Validation of the GH5_5A architecture and activity of its catalytic domain

**Fig. 2:**
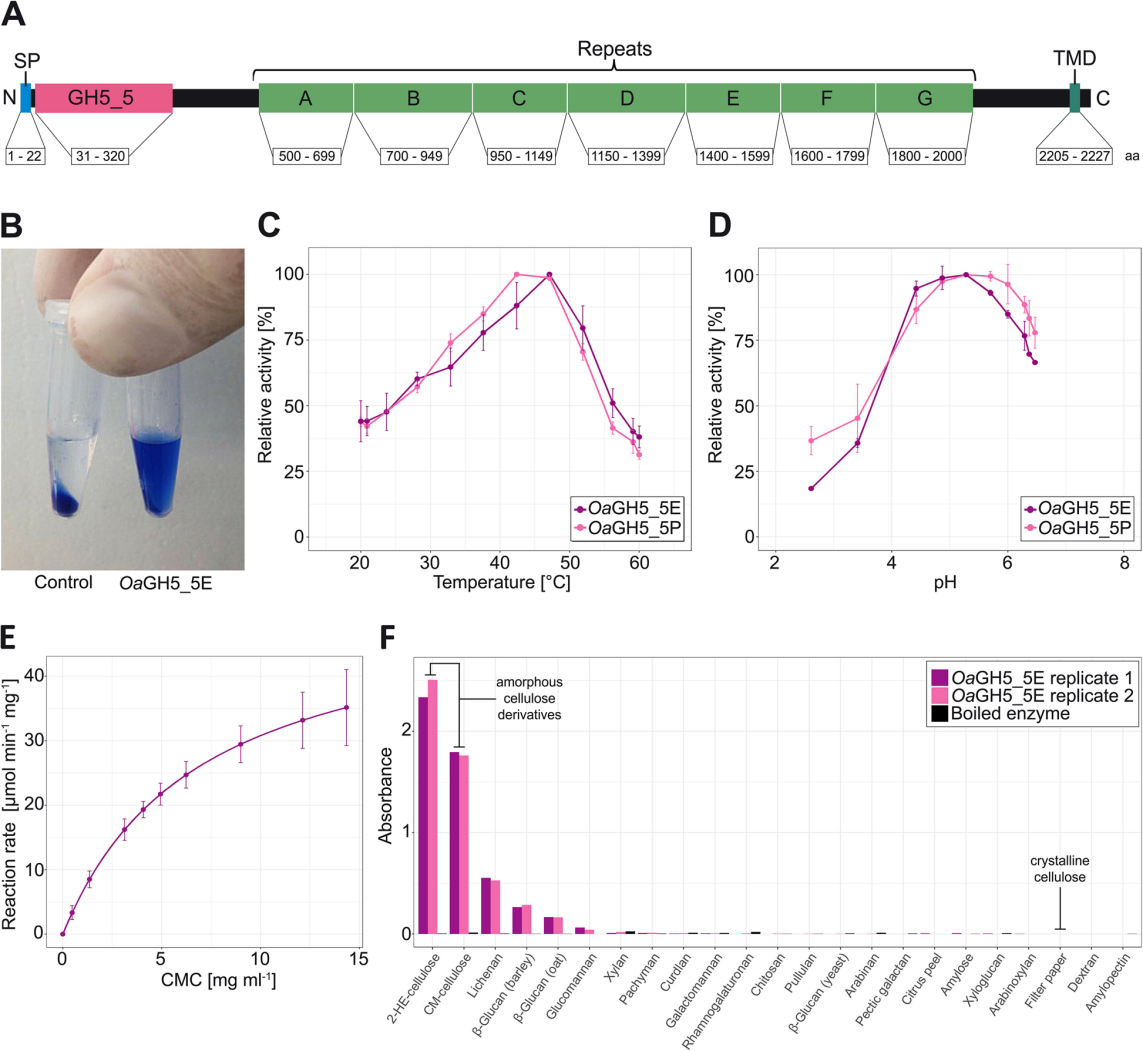
Domain structure of GH5_5A and enzymatic activity of its recombinant catalytic domain. **A** Predicted domain structure of the GH5_5A protein with boundaries of relevant domains (amino acid numbers from the start). **B** Degradation of chromogenic carboxymethyl cellulose (Azo-CM-Cellulose) by *Oa*GH5_5E results in a blue supernatant after centrifugation. **C** Relative activity of two recombinant GH5_5 domains over a temperature range (mean and standard deviation; *n* = 6). **D** Relative activity of two recombinant GH5_5 domains over a pH range (mean and standard deviation; *n* = 6). **E** Michaelis-Menten kinetics of *Oa*GH5_5P with CMC-Na as substrate (mean and standard deviation; *n* = 6). **F** Activity of the recombinant protein *Oa*GH5_5E on 23 chromogenic carbohydrates as determined by absorbance measurements after the GlycoSpot assay. Two replicates and boiled enzyme (as negative control) have been used

Based on transcriptome assemblies [27], the relatively large hypothetical protein (2248 amino acids; approx. 226 kDa) was predicted to contain an N-terminal Sec/SPI signal peptide, a GH5_5 catalytic domain, internal repeats of unknown function (weakly annotated as CBM2) and a C-terminal transmembrane domain (Fig. 2A). We applied PCR to cDNA of *Orciraptor* with five overlapping primer sets that span most of the predicted gene sequence of GH5_5A (Fig. 2A). All PCRs gave products of the expected sizes and the sequences retrieved from Sanger sequencing verified the existence of the GH5_5A protein in its predicted form (gene sequence available at GenBank under the accession OP778256). We used a codon-optimised sequence of the GH5_5 catalytic domain (*Oa*GH5_5) for expression in two hosts, *Escherichia coli* and *Pichia pastoris*. After cloning, protein production and purification, we checked for the predicted molecular weight of *Oa*GH5_5 (37 kDa) by SDS-PAGE (Additional file 1: Fig. S2). The proteins *Oa*GH5_5E (from *E. coli*) and *Oa*GH5_5P (from *P. pastoris*) were subjected to different enzymatic assays to assess their activity, kinetics and substrate specificity. Both proteins showed clear activity on chromogenic carboxymethyl cellulose (Azo-CM-Cellulose), a dye-linked, water-soluble substrate for endocellulases [29]. The presence of the enzymes resulted in blue supernatants (with soluble breakdown products) after precipitation of the substrate (Fig. 2B). The temperature optima were in the range of 42–47 °C, and the activity dropped drastically at > 50 °C (Fig. 2C). The optimum pH was about 5.3 for both proteins, which however showed a high relative activity (> 85%) in a range of 4.3–6.0 (Fig. 2D). Temperature stability of the GH5_5 domain was low, as the protein *Oa*GH5_5E showed almost no activity after heat treatment with ≥ 60 °C for 10 min (Additional file 1: Fig. S3). We also performed Michaelis-Menten kinetics with the 3,5-dinitrosalicylic acid (DNS) assay [30] and carboxymethyl cellulose sodium salt (CMC-Na) as substrate (Fig. 2E). The kinetic parameters of *Oa*GH5_5P were *k*_cat_ = 24.1 ± 1.75 s^−1^, *K*_m_ = 6.90 ± 0.69 mg ml^−1^ and *V*_max_ = 52.05 U min^−1^. To assess the substrate specificity of the GH5_5 domain, we utilised the microtiter plate-based GlycoSpot assay with 23 chromogenic carbohydrates [31]. The highest activities of *Oa*GH5_5E were measured for the cellulose derivatives 2-hydroxyethyl cellulose and carboxymethyl cellulose, while there was no detectable activity on the crystalline cellulose in filter paper (Fig. 2F)—a typical pattern for endo-acting cellulases (see Discussion for details). Other substrates with clear degradation were lichenan and the glucans from barley and oat.

### Cellular localisation of the GH5_5A endocellulase

We used a custom polyclonal antibody (pAb) produced in rabbits against the non-denaturated *Oa*GH5_5P protein to localise the GH5_5A endocellulase in *Orciraptor*. In western blots with whole cell lysate of *Orciraptor*, the affinity purified anti-GH5_5 pAb detected a single band of the right size (sometimes two bands due to potential degradation of the GH5_5A protein), suggesting specific binding to the target protein (Additional file 1: Fig. S4). To follow the distribution of the GH5_5A endocellulase during the feeding process, we performed immunostainings on free, gliding flagellates and on cells at different time points after the addition of algae (a.a.a.). The chosen time points (30 min, 1 h, 2 h and 4 h a.a.a.) correlated well with distinct cellular stages from attachment to phagocytosis.

**Fig. 3:**
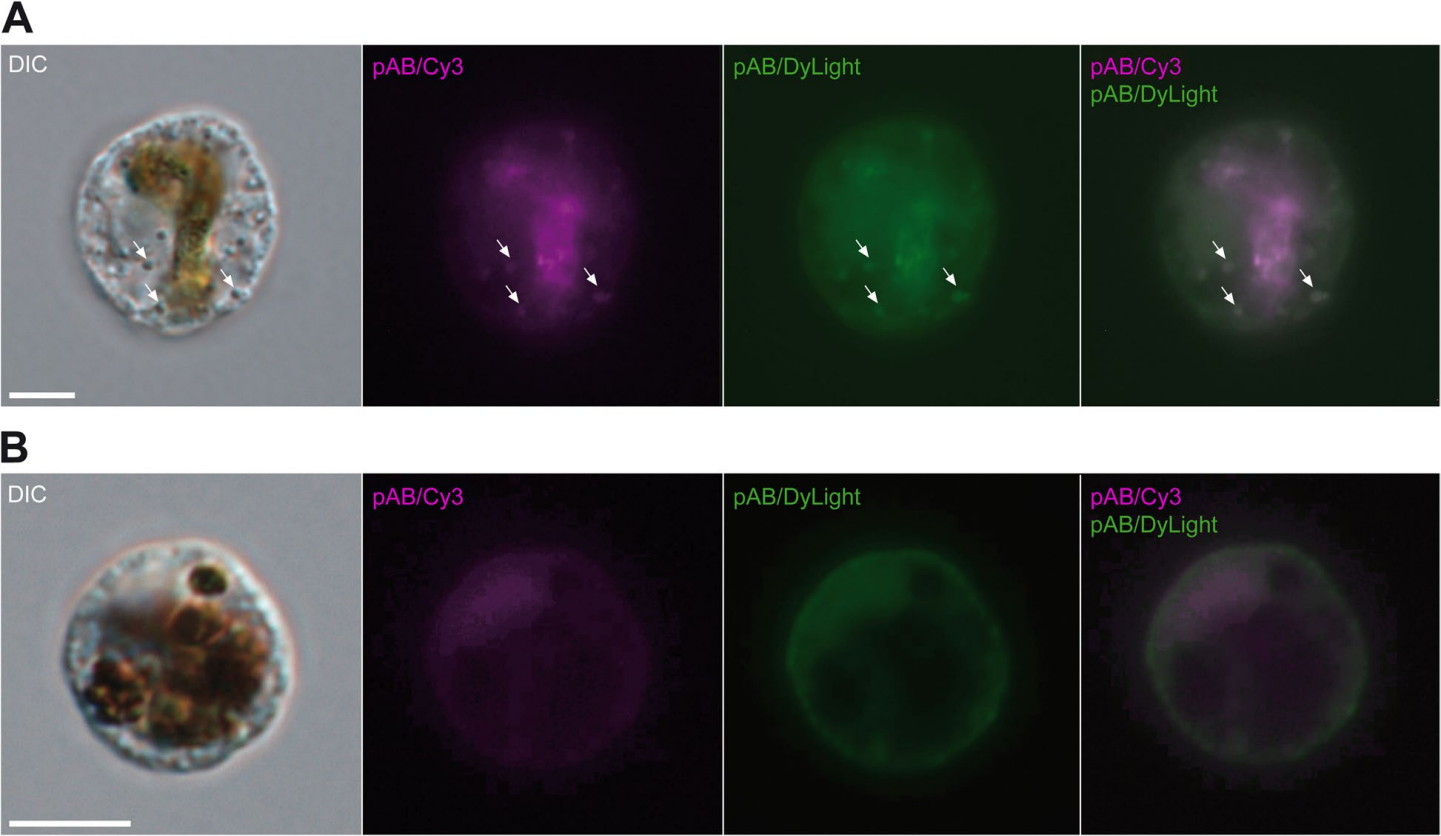
Free *Orciraptor* flagellates stained with the anti-GH5_5 pAb and two secondary antibodies. **A** Weak signals of both secondary antibodies (Cy3 and DyLight® 488) co-localise in cytoplasmatic granules (arrows). Food remnants show some fluorescence as well. **B** Cell prepared with the same protocol but without primary pAb does not contain any granules that fluoresce in both channels. All fluorescence micrographs represent single focal planes captured by widefield microscopy. Scale bars = 5 μm

**Fig. 4:**
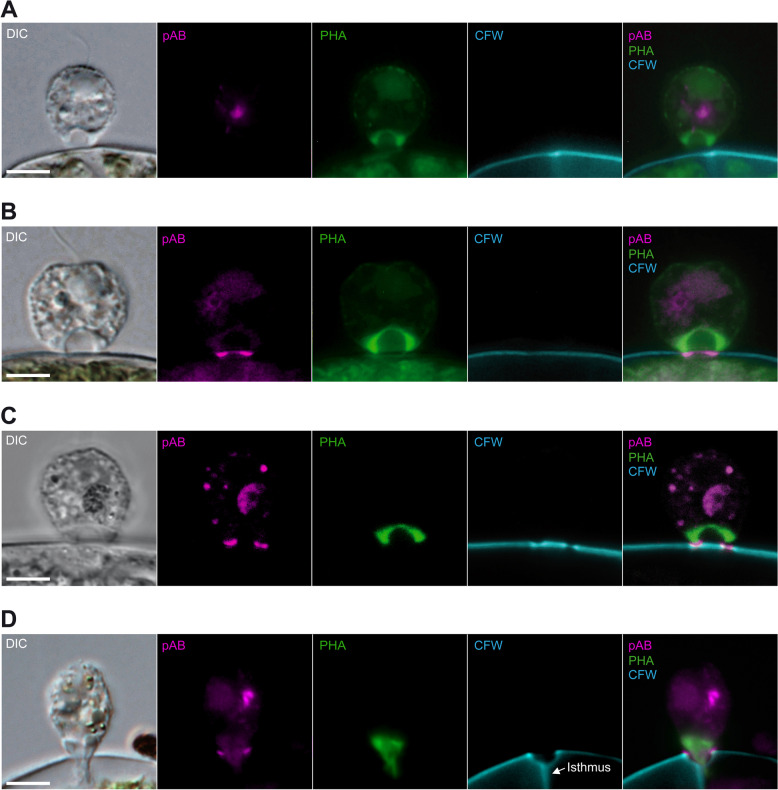
Distribution of the anti-GH5_5 pAb (pAb), F-actin (PHA) and cellulose (CFW) during different stages of attack by *Orciraptor agilis* on *Actinotaenium* cf. *silvae*-*nigrae*. **A** Attached *Orciraptor* cell with lysopodium, 10 min a.a.a. **B** Attached *Orciraptor* cell with lysopodium and pronounced GH5_5 signal at the algal cell wall, 1 h a.a.a. **C** Attached *Orciraptor* cell with lysopodium and pronounced GH5_5 signal at the indented algal cell wall (and at some intracellular structures), 2 h a.a.a. **D ***Orciraptor* cell with pseudopodium extending into the *Actinotaenium* cell (the vertical line visible in the CFW-stained algal cell is out-of-focus fluorescence of the isthmus). The GH5_5 signal localises to the hole in the algal cell wall (and to some intracellular structures), 4 h a.a.a. Fluorescence micrographs in **A**, **B** and **D** represent single focal planes captured by widefield microscopy, in **C** captured by confocal laser scanning microscopy. Scale bars = 5 μm

The free, gliding cells contained small, colourless granules at various number with a very weak anti-GH5_5 signal. This was supported by preparations with two secondary antibodies with different fluorophores (Cy3 and DyLight® 488), both localising to the same granules (arrows; Fig. 3A). Controls without primary antibody and with pre-immune serum showed no stained granules (Fig. 3B). *Orciraptor* cells in early attack (10 min a.a.a.) were attached to the algal cell wall and exhibited an F-actin-rich lysopodium with its typical cup-shaped morphology (Fig. 4A). There were no visible irregularities (gaps) in the Calcofluor White staining of the algal cell wall. The latter thus appeared to be intact. Some *Orciraptor* cells showed a distinct anti-GH5_5 signal in the cell body (Fig. 4A), for example, in the form of granules similar to those observed in the gliding flagellates but with much stronger fluorescence. The presence of these granules varied strongly between individual cells, but a relative quantification of granule-containing cells revealed pronounced differences between the studied stages (see below). Here, we first focus on processes at the algal surface. During a later stage (1 h a.a.a.), the morphology of the *Orciraptor* cells appeared unchanged and the algal cell walls still intact. However, there was a strong and discrete anti-GH5_5 signal confined to the narrow attachment zone of *Orciraptor*, i.e. the contact points of the lysopodium and the algal cell (Fig. 4B). As shown by confocal microscopy data, the GH5_5 endocellulase was localised in form of a ring that corresponds to *Orciraptor’s* perforation pattern (Additional file 2: Video S1). About one hour later (2 h a.a.a.), the algal cell walls exhibited clear indentations of different depths as visualised by the Calcofluor White staining (Fig. 4C). These indentations clearly co-localised with a strong GH5_5 signal (Fig. 4C); they may correspond to the erosion zones observed in our scanning electron micrographs (Fig. 1G, H). In the samples prepared 4 h a.a.a., most algal cell walls exhibited complete perforations with removed or displaced lids, and the *Orciraptor* cells were captured in the process of prey cell invasion. Their pseudopodia run through the cell wall hole into the algal cell, while the cell body stayed outside. A clear GH5_5 signal was present at the margins of the hole, but not in or at the pseudopodia of *Orciraptor* (Fig. 4D).

**Fig. 5:**
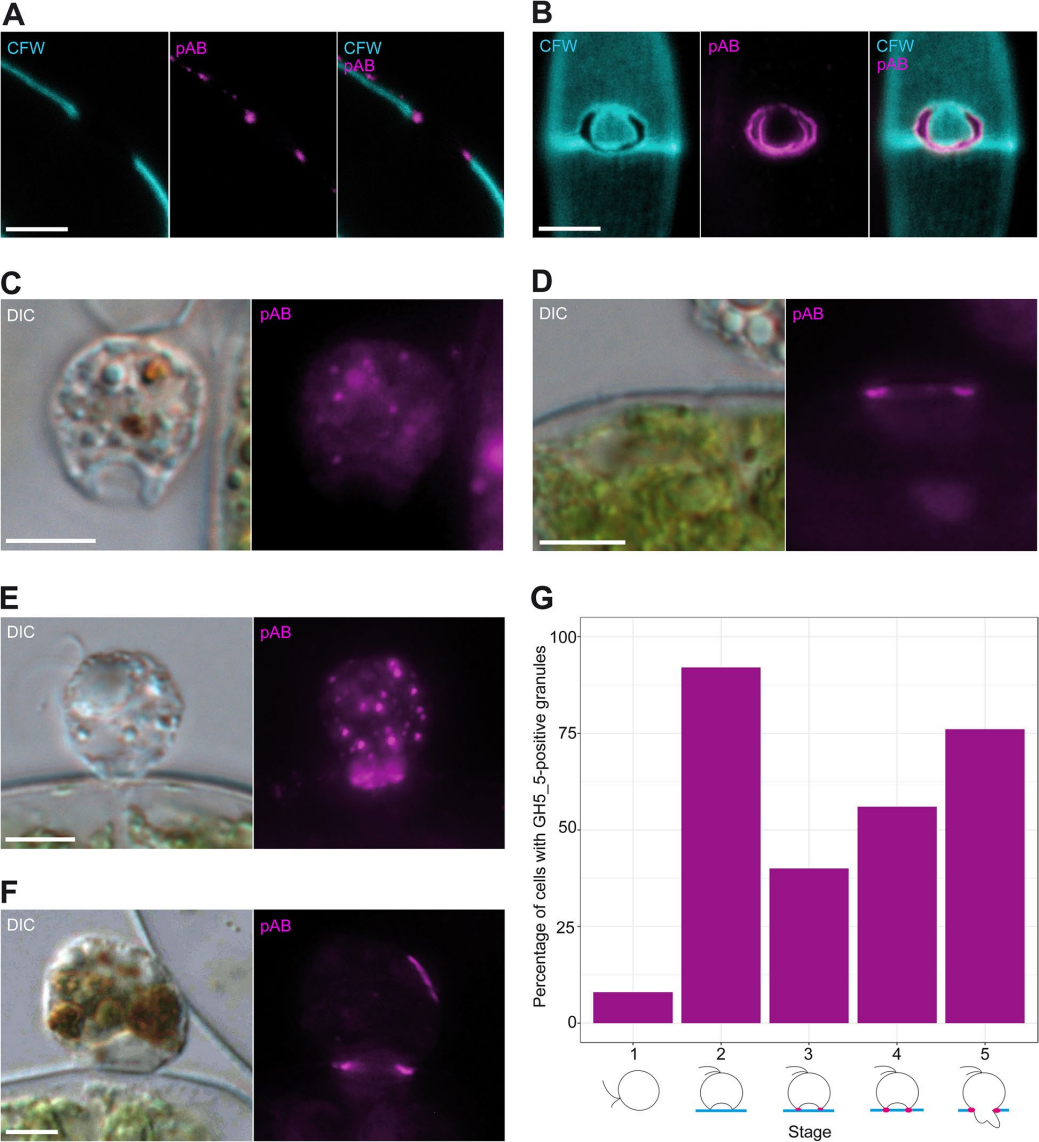
Distribution of GH5_5 signal on algae and dislodged *Orciraptor* cells and occurrence of GH5_5-positive granules over *Orciraptor’s* life history. **A** Optical cross section of an empty *Actinotaenium* cell with clear GH5_5 signal (pAb) at the margin of the hole (CFW stained cellulose in the algal wall). **B** C-shaped perforation with attached lid (in top-view, visualised by CFW fluorescence) and pronounced anti-GH5_5 signal (pAb) at the margins; Z-projection. **C** Attacking *Orciraptor* cell (experimentally dislodged from the alga, 1 h a.a.a.) without GH5_5 signal at the lysopodium. **D*** Actinotaenium* cell which has been experimentally freed from attacking *Orciraptor* cell displays a strong, ring-like GH5_5 signal (in cross section) at its surface. **E** Attacking *Orciraptor* cell with numerous GH5_5-positive granules in the cytoplasm; fluorescence micrograph is a Z-projection. **F** Attacking *Orciraptor* cell without GH5_5-positive granules of high intensity. **G** Percentage of *Orciraptor* cells with GH5_5-positive granules in the cytoplasm at five different time points (stages) during the feeding act: (1) free flagellates before feeding; (2) attached cell without GH5_5 signal at contact zone, 30 min a.a.a.; (3) attached cell with GH5_5 signal at contact zone but no visible cell wall alterations, 1 h a.a.a.; (4) attached cell with GH5_5 signal at contact zone and clear signs of cell wall lysis, 2 h a.a.a.; (5) *Orciraptor* with invading pseudopodia, 4 h a.a.a. (25 cells per stage analysed). Fluorescence micrographs in **A**, **C**, **D** and **F** represent single focal planes, those in **B** and **E** are Z-projections. All images except **A** (confocal laser scanning microscopy) were captured by widefield microscopy. Scale bars = 5 μm

Observations on emptied algal cells from depleted *Orciraptor* cultures revealed that a strong GH5_5 signal remained at the margins of the cell wall holes and lids (Fig. 5A, B) after *Orciraptor* left. Based on the gene structure of the GH5_5A protein (with a signal peptide and transmembrane domain), Gerbracht et al. suggested that the protein could potentially be tethered to the external side of *Orciraptor’s* plasma membrane and take part in contact digestion [27]. To test whether the GH5_5A endocellulase also localises to the cell surface of *Orciraptor*, attacking cells (1 h a.a.a.) were prepared for immunolocalisation and then forcefully dislodged from the algal cells by pipetting. These dislodged cells exhibited the characteristic morphology during attack, but no GH5_5 signal at the lysopodium (Fig. 5C). Instead, strong, ring-shaped signals were found on the algal cells of the same sample (Fig. 5D).

### Intracellular occurrence of GH5_5-positive granules

The presence and number of intracellular, clearly GH5_5-positive granules varied among *Orciraptor* cells in all preparations. To test whether these granules correlate with certain life history stages of *Orciraptor*, we quantified the relative number of *Orciraptor* cells with granules over the following stages: gliding cells (stage 1), cells during early attack without GH5_5 signal at the attachment site (stage 2), cells with GH5_5 signal at the attachment site but no visible alterations of the algal wall (stage 3), cells with GH5_5 signal at the attachment site and indentations in the algal wall (stage 4) and cells with extended pseudopodia during invasion (stage 5). Only granules with a GH5_5 signal comparable to that found in the attachment site of attacking cells were considered (see Fig. 5E). No visible intracellular signal under the same imaging settings (exposure time, gain) was defined as the absence of clearly GH5_5-positive granules (see Fig. 5F). As shown by the counting results (25 cells per stage), the relative number of *Orciraptor* cells with GH5_5-positive granules varied markedly between the stages (Fig. 5G). Only 8% of the gliding cells exhibited fluorescent granules, while cells from stage 2 (early attack), with 92%, showed a much higher frequency. The other three stages had intermediate values with 40% (stage 3), 56% (stage 4) and 76% (stage 5). The cells of all stages exhibited no distinct intracellular distribution of the granules and the presence of granules did not correlate with the presence of food remnants from the previous feeding act.

### Effect of the anti-GH5_5 pAb on *Orciraptor’s* feeding process

**Fig. 6:**
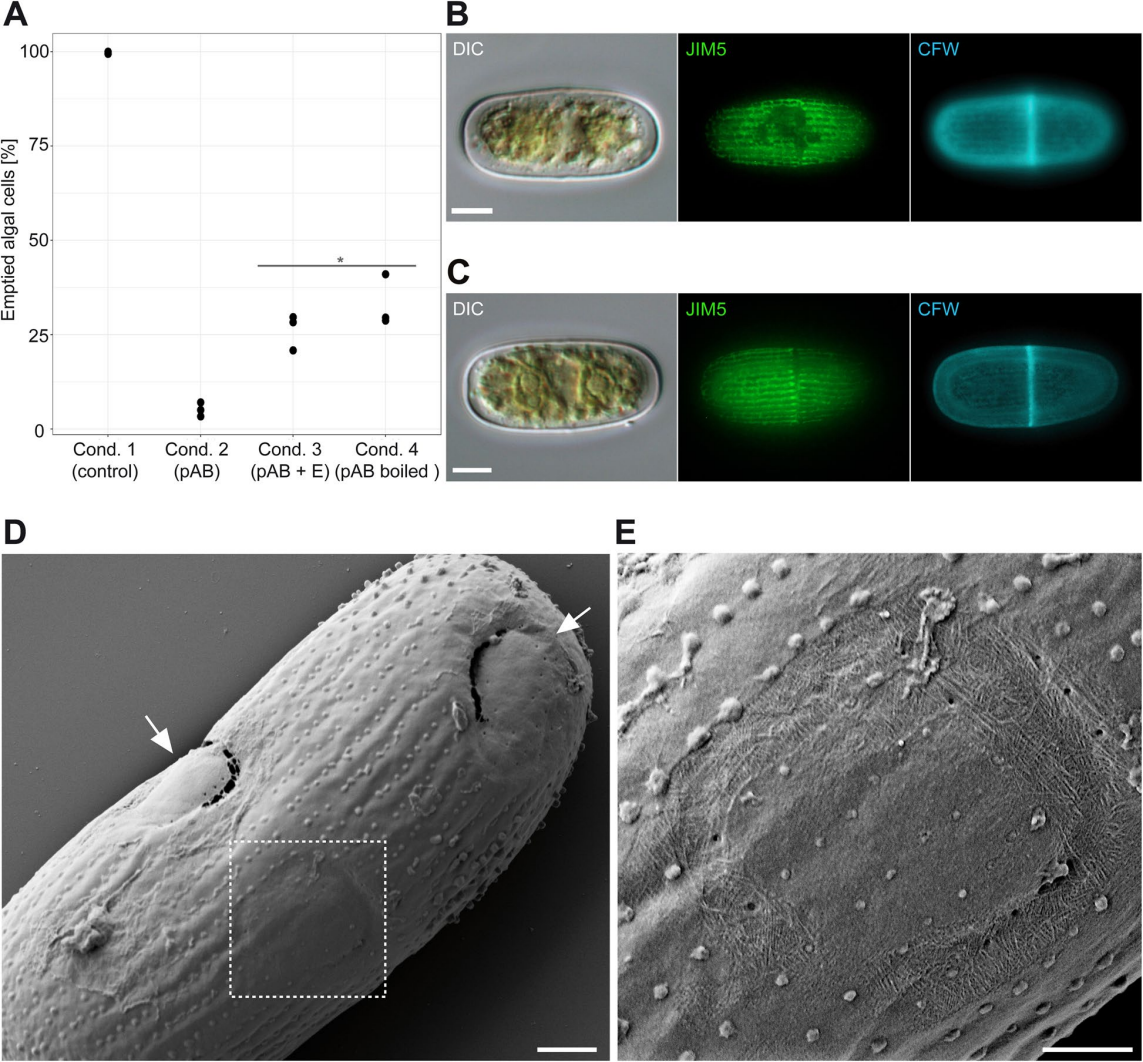
Effect of the anti-GH5_5 pAb on the feeding process of *Orciraptor agilis*. **A** Percentage of emptied algal cells in usual cultures (condition 1) and those supplemented with the anti-GH5_5 pAb (condition 2), with the anti-GH5_5 pAb plus the *Oa*GH5_5P enzyme (condition 3), and the boiled anti-GH5_5 pAb (condition 4). **B** Pectin and cellulose distribution in an *Actinotaenium* cell from condition 2 visualised by fluorescence staining (JIM5 = pectin; CFW = cellulose). **C** Pectin and cellulose distribution in an *Actinotaenium* cell which did not have contact to *Orciraptor* (JIM5 = pectin; CFW = cellulose). **D*** Actinotaenium* cell from condition 2 with at least one attempted attack (dashed rectangle) and two incomplete perforations (arrows), SEM. **E** Close-up of the attempted perforation shown in **D** reveals large areas of exposed cellulose microfibrils, SEM. Fluorescence micrographs in **B** and **C** represent Z-projections of image data from widefield microscopy. Scale bars in **B**, **C** = 5 μm, in **D** = 2μm, in **E** = 1 μm

To assess the importance of the GH5_5A endocellulase for the feeding process in *Orciraptor*, we tested the effect of the anti-GH5_5 pAb on live cells. The immunoglobulins of the pAb should specifically bind to epitopes of the GH5_5 catalytic domain of the native endocellulase and thereby interfere with the cleavage of the substrate. Gliding *Orciraptor* cells were exposed to (1) normal culture medium as a control, (2) the pAb in culture medium, (3) the pAb + *Oa*GH5_5P in culture medium and (4) boiled pAb in culture medium (see the “Methods” section for details). Under all conditions, the flagellates displayed their typical motility and readily attacked freeze-killed *Actinotaenium* cells. After 14 h, we quantified the percentage of emptied algal cells in three replicates of each condition (*n* ≥ 200 cells per sample). In the control, almost 100% of the algal cells were emptied, while the pAb solution (condition 2) drastically reduced the feeding success of *Orciraptor* to approximately 5% (Fig. 6A). Both the addition of recombinant *Oa*GH5_5P to the pAb (condition 3) and boiling of the pAb solution before the experiment (condition 4) increased the feeding success compared to condition 2, with > 25% emptied algal cells in both cases. According to the post-hoc Tukey test (with a significance threshold of *p*-value < 0.05), all conditions were significantly different (*p*-values ≤ 1.37e^−3^) except conditions 3 and 4 with a *p*-value of 0.278 (Fig. 6A).

Time-lapse imaging of *Orciraptor* cells under condition 2 revealed that they attacked as normal but detached from the algae after several hours without having fed. To check for any cell wall alterations in the algae, we applied fluorescence staining methods for pectin (JIM5 antibody) and cellulose (Calcofluor White). *Actinotaenium* cells from condition 2 frequently displayed a patchy degradation of the pectin layer, while their cellulosic wall components appeared intact (Fig. 6B). This was in stark contrast to algal cells which have not been in contact with *Orciraptor*. They displayed an intact pectin layer with regular longitudinal striations (Fig. 6C). Scanning electron microscopy revealed that algal cells from condition 2 exhibited ring-like surface alterations as well as incomplete perforations, sometimes several per cell (Fig. 6D; arrows). The affected regions exhibited exposed cellulose microfibrils, indicating the removal of pectic substances by *Orciraptor* but impaired degradation of cellulose (Fig. 6E).

## Discussion

In this study, we provide experimental evidence that the highest expressed CAZyme (GH5_5A) of the protoplast feeder *Orciraptor agilis* is involved in the pre-phagocytotic perforation of algal cell walls. Specifically, we assessed the activity of the GH5_5 catalytic domain, localised the native protein in the cellular context and tested the effect of a specific anti-GH5_5 pAb on the biological process. Our new experimental system consisting of *Orciraptor agilis* and the desmid *Actinotaenium* cf. *silvae-nigrae* turned out to be very useful. The unicellular, thick-walled alga let us study *Orciraptor’s* perforation process at higher temporal resolution and enabled a quantification of feeding events.

The recombinant GH5_5 domain was successfully produced in both prokaryotic and eukaryotic expression systems. It showed a clear activity on the cellulose-derived β-1,4-glucans CMC and HEC and to lesser extent on mixed-linkage glucans with β-1,4- and β-1,3-glucosidic linkages (lichenan, glucans from barley and oat). There was no detectable activity on crystalline cellulose (filter paper), which suggests that the GH5_5A protein from *Orciraptor* is an endocellulase [32]. This is consistent with the knowledge about other members of the glucoside hydrolase family 5 subfamily 5 from bacteria and fungi [33–36]. In fact, GH5_5 domains are widespread among bacteria, fungi and diverse protists [27], and our data on the GH5_5 from *Orciraptor agilis* suggest that their enzymatic function might be relatively conserved. However, the limited ability of such enzymes to degrade crystalline cellulose in vitro does not exclude that the native GH5_5A protein could be involved in the breakdown of cellulose microfibrils in algal cell walls. As shown by other studies [37, 38], the fusion of GH5_5 domains with cellulose-binding CBMs enabled these domains to degrade crystalline forms of cellulose. Furthermore, in biological systems CAZymes of different families often work in synergy to degrade crystalline substrates, and the transcriptome of *Orciraptor agilis* contains numerous other factors that may bind and/or act on cellulose [27]. The reaction optima and the affinity of the recombinant GH5_5 domain from *Orciraptor* to CMC are in line with the properties of other GH5_5 endocellulases [34, 39–41]. The broad pH optima in the range of 4–6 match the conditions of *Orciraptor’s* habitats (the acidic waters in moorlands, [2]) very well, suggesting an efficient action of the GH5_5 endocellulase under natural conditions.

The anti-GH5_5 immunostainings of attacking *Orciraptor* cells revealed that the GH5_5A endocellulase localises to the narrow degradation zone in the algal cell wall. This strongly indicates the involvement of this protein in the perforation process. Interestingly, the endocellulase appeared to be absorbed to the algal cell wall, as the GH5_5 signals remained at the perforation sites of emptied algal cells. This could potentially be caused by the repeats found in the GH5_5A protein, which were weakly annotated as CBM2, a cellulose-binding module [27]. Some CBMs are known to bind very strongly, almost irreversibly to their target [42–44]. However, the binding functions of the native GH5_5 protein require future study. Based on the gene structure of the GH5_5A endocellulase (with a signal peptide and transmembrane domain), it was hypothesised that this protein could be tethered to the plasma membrane of *Orciraptor* and take part in contact digestion [27]. Cell surface-bound CAZymes are known, for example, from the “cellulosomes” of cellulose-degrading bacteria and fungi [45–47]; though in these systems, CAZymes are tethered indirectly to the plasma membrane by a protein scaffold. We did not observe any GH5_5 signal at the cell surface of *Orciraptor*, including the cells that have been forcefully dislodged from the alga during attack. Hence, there is no support for contact digestion by membrane-tethered GH5_5A protein, and it remains unclear how exactly *Orciraptor* delivers the endocellulase to the well-defined perforation zone. We cannot exclude that the active part of the GH5_5A protein is split and released from its transmembrane domain at a certain stage. Such processes, for example protease-mediated ectodomain shedding, are known from other, well-studied cellular systems [48]. The presence of GH5_5-positive granules in the cell body of *Orciraptor* points to an intracellular origin and vesicular localisation of the enzyme. We demonstrated that the proportion of cells with these putative vesicles increased drastically during attack as compared to starving cells (from 8 to 92%), suggesting a triggered biosynthesis of the GH5_5A endocellulase after contact with the algae. This aligns very well with the published differential expression data, since the GH5_5A protein and various components for protein biogenesis are highly upregulated in *Orciraptor* during attack [27]. All this suggests that the GH5_5A endocellulase is synthesised de novo for each feeding event. The drop in number of cells with endocellulase vesicles in stage 3 coincides with the presence of the GH5_5-positive ring at the algal surface, and, hence, might relate to the secretion of the produced endocellulase. How exactly this protein is secreted in the well-defined pattern remains unclear, but we hypothesise that actin-related vesicle transport associated with *Orciraptor’s* lysopodium might play a role.

Furthermore, we treated live *Orciraptor* cells with the specific pAb against the catalytic domain of the GH5_5A endocellulase and demonstrated that the pAb drastically reduces the feeding success. As observed in cell wall stainings and SEM micrographs of *Actinotaenium* cells from these samples, the *Orciraptor* cells made numerous attempts to perforate the algal cell walls. For the most part, this resulted only in surface alterations such as the removal of rosettes and pectic substances (probably degraded by other enzymes) or incomplete perforations. This suggests that the anti-GH5_5 pAb effectively inhibits cellulose degradation during *Orciraptor’s* feeding act, while other enzymatic functions remain intact. Interestingly, this inhibition could be mitigated by the addition of the recombinant *Oa*GH5_5P protein as specific competitor and by heat denaturation of the pAb before use. Hence, the inhibiting effect of the pAb may be conferred by specific binding to *Orciraptor’s* GH5_5 domain and not, for example, by the increased protein concentration in the medium. The remaining cellulolytic activity responsible for the incomplete perforations produced in the presence of the anti-GH5_5 pAb might stem from some of the other glycoside hydrolases of *Orciraptor* with potential cellulase function [27]. However, the pronounced effect of the GH5_5-specific pAb observed in our experiments suggests that the GH5_5A endocellulase of *Orciraptor* is a key factor for the dissolution of algal cell walls.

## Conclusions

Based on transcriptome data of the microbial protoplast feeder *Orciraptor agilis*, we selected the highest expressed CAZyme, a putative endocellulase of family GH5_5, for a biochemical and functional characterisation. Three lines of experimental evidence, namely the activity of this enzyme on cellulosic compounds, the localisation of this enzyme at the perforation zone and the interference of anti-GH5_5 antibodies with the feeding process of *Orciraptor*, point to the importance of this extracellular endocellulase in the pre-phagocytotic cell wall perforation. Protoplast feeding is a widespread feeding strategy of phylogenetically diverse, phagotrophic microeukaryotes. On the mechanistic level, it differs drastically from the processes observed in typical predators and osmotrophic parasites. Here, we provide the first evidence that the well-defined cell wall perforations produced by protoplast feeders are caused by enzymatic activity and made the first step towards establishing the cell biological basis of a fascinating, yet poorly understood microbial feeding strategy.

## Methods

### Organisms and cultivation

*Orciraptor agilis* (strain OrcA03) was cultivated as previously described [2]. In short, the food alga *Mougeotia* sp. (strain CCAC 3626) was freeze-killed, suspended in demineralised water, inoculated with *Orciraptor* cells and then kept at 4 °C in the dark. *Mougeotia* sp. (strain CCAC 3626) was grown in the liquid growth medium Waris-H [49] supplemented with 1% (v/v) bacterial standard medium (0.8% peptone, 0.1% glucose, 0.1% meat extract, 0.1% yeast extract in distilled water (w/v); [50]), under artificial light from white LEDs (photon fluence rate 10–30 μmol m^−2^ s^−1^, 14:10 h light–dark cycle) and at 15 °C. *Actinotaenium* cf. *silvae*-*nigrae* (strain CCAC 0140) was grown under the same conditions but in pure Waris-H. Routine microscopy on long-term and experimental cultures was done with the Motic AE2000 inverted microscope (Motic Hong Kong Limited, Hong Kong). The algal strains are available from the Central Collection of Algal Cultures (CCAC) at the University of Duisburg-Essen (https://www.uni-due.de/biology/ccac/), *Orciraptor agilis* (OrcA03) from the corresponding author upon request.

### RNA isolation, cDNA synthesis, PCR and sequencing

Total RNA was isolated with the TRI Reagent® (Merck KGaA, Darmstadt, Germany) from *Orciraptor agilis* as described in Gerbracht et al. [27], and then subjected to digestion with DNase I (New England Biolabs, Frankfurt am Main, Germany) and used for cDNA synthesis with reverse transcriptase (Thermo Fisher, Waltham, Massachusetts, US). The cDNA was subjected to PCR with five pairs of primers (Table 1), which spanned most of the hypothetical GH5_5A gene. The PCR was done with the Phusion™ High-Fidelity DNA Polymerase (Thermo Fisher, Waltham, Massachusetts, US) using the following protocol: Initial denaturation (30 s at 98 °C), then 37 cycles of denaturation (15 s at 98 °C), annealing (30 s, see Table 1 for primer-specific temperatures) and extension (2 min at 72 °C), followed by a final extension step (2 min at 72 °C). PCR products were checked by agarose gel electrophoresis, purified with the NucleoSpin Gel and PCR Clean-up Kit (Takara Bio, Kusatsu, Shiga, Japan) and sequenced by commercial Sanger sequencing (Eurofins Genomics, Ebersberg, Germany) with the primers used for PCR (Table 1).

**Table 1:** Primers used in this study

Name	Sequence (5′ to 3′)	Used annealing temp. (°C)
1F	CTACACTTGCTCCTACGACAGC	65
1R	CCATTGCTGCATTGAGGCGTG	65
2F	CGTATCGGTCTCCGCTAATGC	63
2R	CAGTTTCGATGTTGTTAACTGCTG	63
3F	CCGGTGGCGCTACAACTAC	66
3R	CTGATTGGTGTTGAAATTCACAGTGATC	66
4F	CACTTACTCCTACCACGCCTTG	65
4R	CAAGAGTTAACTGAGCACCAGGGT	65
5F	CAGGCAATGTTTGGACATTTGGTCTAC	67
5R	TTAAGCTGAGTTAGCCATTGCGATGTTATTTTC	67
GH_F	CTGGAAGTTCTGTTCCAGGGTCCGGGAATGGGCGCGCACCTG	68
GH_R	CAGTGGTGGTGGTGGTGGTGCTCGAGTCACAGGGTGGTC	68
GST_F	AACTTTAAGAAGGAGATATACATATGTCCCCTATACTAGGTTATTGG	68
GST_R	GGAACAGAACTTCCAGTTTTGGAGGATGGTCGCCAC	68

### Plasmid construction, expression and purification of *Oa*GH5_5E (from *E. coli*)

The codon optimised GH5_5 domain (Additional file 3) was delivered in a pUC57-Kan vector (Genscript, Piscataway, US) and amplified by PCR with the Phusion™ High-Fidelity DNA Polymerase (Thermo Fisher, Waltham, MA, USA) and the primers GH_F and GH_R (Table 1). Likewise, the sequence encoding for a GST-tag was amplified from *pGEX*-4T-1 with the primers GST_F and GST_R (Table 1). The PCR products were inserted into pET-22b(+) with the NEBuilder HiFi DNA Assembly Cloning Kit (New England Biolabs, Frankfurt am Main, Germany) resulting in a pET22b-GST-*Oa*GH5_5 construct. SHuffle T7 Express cells (New England Biolabs, Frankfurt am Main, Germany) were transformed with the construct by heat shock treatment according to the protocol by Inoue at al [51]. and used for expression of GST-GH5_5 at 15 °C with continuous agitation at 180 rpm after induction by 0.2 mM isopropyl thio-β-D-galactoside (IPTG). After harvest, cells were lysed by sonication on ice in lysis buffer (100 mM Tris-HCl, 150 mM NaCl, 0.1% Triton X-100, cOmplete^TM^ EDTA-free protease inhibitor cocktail (Roche Diagnostics, Mannheim, Germany), 1 μg ml^−1^ DNAse I (New England Biolabs, Frankfurt am Main, Germany); pH 8.0). Cellular debris was pelleted by centrifugation (10,000 *g*, 30 min, 4 °C), and the protein in the supernatant was purified with glutathione sepharose 4B (GE Healthcare, Chicago, Illinois, US) and concentrated with a Vivaspin 20 centrifugal concentrator (5 kDa MWCO; Sartorius AG, Goettingen, Germany). The purified protein was quantified according to Bradford [52] using bovine serum albumin as a standard and checked by sodium dodecyl sulphate polyacrylamide gel electrophoresis (SDS-PAGE) with 10% acrylamide.

### Plasmid construction, expression and purification of *Oa*GH5_5P (from *P. pastoris*)

The GH5_5 domain amplified from pUC57-*Oa*GH5-5 (details above) was inserted into pGAPZα (Invitrogen, Waltham, MA, USA) with the NEBuilder HiFi DNA Assembly Cloning Kit (New England Biolabs, Frankfurt am Main, Germany). The resulting expression plasmid pGAPZα-*Oa*GH5_5 also contained an N-terminal yeast α-secretion factor and C-terminal His_6_tag. The plasmid was then linearized with *AvrII* (New England Biolabs, Frankfurt am Main, Germany) and transformed into *P. pastoris* strain KM71H (Invitrogen, Waltham, MA, USA) by electroporation according to the EasySelect™ Pichia Expression Kit (Invitrogen, Waltham, MA, USA). Transformants were selected on YPDS (1% yeast extract, 2% peptone, 18.2% sorbitol, 2% agar, 2% dextrose) plates containing 100 μg ml^−1^ Zeocin™ (Alfa Aesar by Thermo Fisher Scientific, Kandel, Germany) and used for enzyme production in agitated (200 rpm) large batch cultures with YPD medium (1% yeast extract, 2% peptone, 2% dextrose) supplemented with 100 mM potassium phosphate buffer (pH 6.0). After growth for 3 days at 30 °C, protein was purified from the culture supernatant with Ni-NTA Agarose (Cube Biotech, Monheim, Germany) and transferred to PBS with 20% glycerol using a PD-10 Desalting Column (GE Healthcare, Chicago, IL, USA). The protein was quantified according to Bradford [52] using bovine serum albumin as a standard and checked by SDS-PAGE with 15% acrylamide.

### Enzyme assays and kinetics

The cellulolytic activity of protein samples was determined with chromogenic, solubilised carboxymethyl cellulose, Azo-CM-Cellulose (Megazyme Ltd., Bray, Wicklow, Ireland), according to the manufacturer’s instructions, but with minor modifications: 40 μl protein solution (0.1 mg ml^−1^) was supplemented with 10 μl sodium citrate buffer (1 M, pH 5.5), mixed with 50 μl Azo-CM-Cellulose substrate solution (2%) and incubated at 40 °C. The reaction was stopped after 30 min by adding 250 μl precipitating solution (40 g L^−1^ sodium acetate, 4 g L^−1^ ZnCl_2_, 80% ethanol) and then centrifuged at 17,000 *g* for 10 min. The absorbance of the clear supernatant, which correlates with cellulolytic activity, was determined at 590 nm with a Sunrise™ microplate reader (Tecan, Crailsheim, Germany). To determine the temperature optimum of the cellulase samples, the Azo-CM-Cellulose assay was conducted in a Biometra TOne thermocycler (Analytik Jena GmbH, Jena, Germany) with its gradient function (20–60 °C). The thermostability of the enzyme samples was tested by measuring the enzymatic activity after heat treatment (60, 70, 80 and 90 °C) for 10 min. The pH optimum of the enzyme samples was determined by performing the Azo-CM-Cellulose assay in sodium citrate buffer (50 mM; pH 4.8) adjusted to different pH values. The assays for temperature and pH optima were conducted with six replicates.

Michaelis-Menten kinetics on cellulase samples were performed by quantifying the reducing ends of sugars released from carboxymethyl cellulose sodium salt (CMC-Na, Sigma-Aldrich, Darmstadt, Germany) with the 3,5-dinitrosalicylic acid (DNS) assay [30]. The reaction mix of 250 μl total volume contained the *Oa*GH5_5P enzyme (4.18 mg L^−1^), sodium citrate buffer (10 mM, pH 5.5) and CMC-Na at varying concentration (0–15 mg ml^−1^). After incubation for 30 min at 40 C°, the reactions were stopped by adding 250 μl DNS reagent (0.5% DNS, 0.4 M NaOH, 30% potassium sodium tartrate) and then incubated at 100 °C for 5 min. The samples were then cooled on ice and their absorbance was determined at 540 nm with a Sunrise™ microplate reader (Tecan, Crailsheim, Germany). The concentration of the reducing ends of the released sugars was quantified using a dilution series of glucose as standard. The *K*_m_ value was determined by nonlinear regression of the Michaelis–Menten equation with the GraphPad prism 9.0 software (GraphPad Software, San Diego, California, US). The assay for the Michaelis-Menten kinetics was conducted with six replicates.

To assess the substrate specificity of the GH5_5 domain, a microplate-based GlycoSpot assay (GlycoSpot, Søborg, Denmark) with a custom layout of 23 chromogenic carbohydrate samples was used (for substrates see Fig. 2F) [31]. The assay was performed according to the manufacturer’s instructions. In brief, 200 μl activation solution was added to each well, incubated for 15 min and then removed by centrifugation (2700 *g*, 10 min). The enzyme (*Oa*GH5_5E) was diluted in reaction buffer (50 mM sodium acetate, pH 4.6) to a final concentration of 50 μg ml^−1^, added to the wells of the GlycoSpot microplate and incubated at 42 °C for 1 h, and then at room temperature at 150 rpm overnight. One microplate allowed from three enzyme samples, one of which was boiled before the assay (95 °C, 5 min) and served as negative control. After incubation, the microplate was centrifuged (2700 *g*, 10 min), and the absorbance of the flow-throughs was determined at 595 and 517 nm (depending on the dye linked to the carbohydrate) with a Sunrise™ microplate reader (Tecan, Crailsheim, Germany).

### Antibody generation and validation

A custom polyclonal antibody (pAb) of the IgG type was generated in a rabbit against the non-denatured protein *Oa*GH5_5P by Davids Biotechnologie GmbH (Regensburg, Germany). We obtained pre-immune serum (day 0), test serum (day 35) and an affinity purified pAb (day 63). The concentration of the pAb (0.95 mg ml^−1^) was determined by Davids Biotechnologie GmbH with an enzyme-linked immunosorbent assay (ELISA). To check the specificity of the pAb, we performed western blots with *Orciraptor agilis* whole cell lysate. A densely grown *O. agilis* culture was harvested by centrifugation at 100 *g* for 10 min and mixed with lysis buffer (50 mM Tris/HCl, 1mM EDTA, 150 NaCl, 0.1% Nonidet-T40 and cOmplete^TM^ protease inhibitor cocktail; pH 8). The cell lysate was run on a SDS-PAGE (10% acrylamide). The separated proteins were transferred onto a polyvinyl difluoride (PVDF) membrane by electroblotting for 1 h in transfer buffer (48 mM Tris base, 39 mM glycine, 20% methanol; pH 8.3). The membrane was then blocked in 5% non-fat dry milk in TBS-T (Tris-buffered saline supplemented with 0.1% Tween 20) for 1 h, and then incubated with the anti-GH5_5 pAb (1:1000 in 5% non-fat dry milk solution) under constant agitation at 4 °C overnight. The membrane was washed three times with TBS-T, incubated with a horseradish peroxidase-conjugated anti-rabbit secondary antibody produced in goat (1:20,000; Agrisera, Vännäs, Sweden) and again washed three times with TBS-T for 5 min. Immunodetection on the membrane was carried out with the SuperSignal™ Chemiluminescent Substrate (Thermo Fisher, Waltham, MA, US) using an ImageQuant LAS4000 imaging system (GE Healthcare, Chicago, IL, USA).

### Immunolocalisation by fluorescence microscopy

The immunolocalisation in *Orciraptor* cells was performed as described by Busch and Hess [28] with minor modifications. Algal cells (*Actinotaenium* cf. *silvae-nigrae*, *Mougeotia sp.*) were stained with Calcofluor White and washed with demineralised water before freeze-killing. After thawing, the algal cells were sonicated (30 s), washed with demineralised water and added to a culture of gliding *Orciraptor agilis* cells. The culture was incubated for a defined period of time (see Results) and then fixed with 4% formaldehyde and 0.05% glutaraldehyde in MT buffer (30 mM HEPES, 15 mM KCl, 5 mM EGTA, 5 mM MgSO_4_; pH 7) for 10 min at room temperature. In some samples fixed 1 h a.a.a., the *Orciraptor* cells were deliberately dislodged from the *Actinotaenium* cells by repeated pipetting. After gentle centrifugation (100 *g*, 15 min) and washing with distilled water, the cells were applied to coverslips coated with poly-L-lysine (Sigma-Aldrich, Darmstadt, Germany), and let settle for about 30 min. The coverslips were then centrifuged (1000 *g*, 10 min) to firmly attach the cells and washed with PBS for 10 min. The cells were permeabilised with 0.25% Triton X-100 in PBS for 10 min, washed (PBS, 10 min), blocked with 1 x Roti®Immuno-block (Carl Roth, Karlsruhe, Germany) in PBS for 10 min and incubated with the anti-GH5_5 pAb (1:100 in 0.1% blocking solution) for 30 min at room temperature. For same samples, the pre-immune serum was used as a negative control (applied instead of the primary pAb). After thorough washing and blocking (see above), the cells were incubated with a secondary anti-rabbit IgG (1:500 in PBS) for 30 min at room temperature. We used a Cy3-conjugate and a DyLight® 488-conjugate both produced in goat (Jackson Immuno Research, Cambridge, UK), sometimes in combination. The coverslips were then washed in PBS, incubated with PromoFluor-488 Premium fluorescent phalloidin (0.15 μM in PBS with 1% BSA; PromoCell GmbH, Heidelberg, Germany) for 10 min, washed again and mounted on microscope slides with SlowFade^TM^ Antifade Mountant (Invitrogen, Waltham, MA, USA). The samples were imaged with the ZEISS Axio Observer 7 inverted microscope equipped with the objective lens Plan-Neofluar 100×/1.3, DIC optics, and the Axiocam 512 colour (Carl Zeiss, Oberkochen, Germany). The ZEISS Colibri 5 LED illumination system (RGB-UV) and the following filter sets were used for fluorescence imaging: 43 HE Cy3 (excitation 550/25, emission 605/70), 38 Endow GFP (excitation 470/40, emission 525/50) and 96 HE BFP (excitation 390/40, emission 450/40). Some samples were also imaged with a Leica TCS SPE (SP8) confocal laser scanning microscope using excitation wavelength of 405, 488 and 561 nm, respectively, and the Leica LasX software (Leica Microsystems, Wetzlar, Germany). The z-stacks were recorded with a step size of 0.25 μm, and the data processed with the Fiji software [53] and Photoshop CS4 (Adobe Inc., CA, USA). For each of the five stages as defined in the results (gliding flagellates and 30 min, 1 h, 2 h and 4 h a.a.a.), at least three separate immunolabelling experiments were conducted and analysed.

### Relative quantification of cells with GH5_5-positive granules

To quantify the fraction of *Orciraptor* cells with GH5_5-positive granules before and during the feeding act, a culture (30 ml) was spiked with dead *Actinotaenium* cells. Just before this moment and after defined periods of time during the feeding process (30 min, 1 h, 2 h and 4 h a.a.a.), subsamples of 2 ml were removed from the culture and subjected to immunolabelling of the GH5_5 domain as described above. A minimum of 25 cells per subsample were carefully analysed for the presence of GH5_5-positive granules with the Leica TCS SPE (SP8) confocal laser scanning microscope (details above).

### Scanning electron microscopy

Algal cells were pelleted by centrifugation (1500 *g*, 10 min), resuspended in distilled water and pipetted on coverslips coated with poly-L-lysine (Sigma-Aldrich, Darmstadt, Germany). After 20 min, the cells were centrifuged on the coverslips (1000 *g*, 10 min), and then dehydrated in a graded series of ethanol (50%, 96%, 100%; 5 min each step), transferred to hexamethyldisilazane (Carl Roth, Karlsruhe, Germany) and incubated for 10 min. After an exchange of hexamethyldisilazane the samples were air-dried, sputter-coated with gold and imaged with a ZEISS Neon 40 scanning electron microscope (secondary electron detector, 2.5 kV acceleration voltage; Carl Zeiss, Oberkochen, Germany).

### Anti-GH5_5 pAb assay on live cells

The GH5_5 pAb was concentrated by centrifugation (3400 *g*, 10 min) in Vivaspin® 20 Ultrafiltration units (5000 MWCO, PES, Sartorius AG, Goettingen, Germany) and then washed three times with distilled water with the same system. The following experimental cultures (conditions) were set up in triplicates: Condition 1: *O. agilis* in normal medium (control). Condition 2: *O. agilis* in diluted anti-GH5_5 pAb (1:100). Condition 3: *O. agilis* in diluted anti-GH5_5 pAb (1:100) and diluted *Oa*GH5_5P protein (0.01 mg ml^−1^), both mixed 1 h prior to testing. Condition 4: *O. agilis* in diluted anti-GH5_5 pAb (1:100) which was boiled before use (90 °C, 10 min). After acclimatisation of the *Orciraptor* cells in these solutions for 20 min, freeze-killed *Actinotaenium* cells were added, and the cultures (kept at room temperature) were regularly checked by light microscopy. About 14 h later, the cultures were harvested and prepared for the quantification of emptied algal cells, SEM and cell wall stainings. A minimum of 200 *Actinotaenium* cells (emptied and full) were counted in six separate images taken from each of three technical replicates (= cultures) to determine the percentage of emptied algal cells. Significant differences in the percentage of emptied algal cells between the conditions were determined with Tukey’s post-hoc test [54]. The cells for SEM were fixed with 2.5% glutaraldehyde in HEPES buffer (pH 7) for 10 min and prepared as described above. The cells for cellulose and pectin visualisation were processed as described above (immunolocalisation) but treated with the JIM5 antibody against homogalacturonan (1:10, 1 h; Megazyme Ltd., Bray, Wicklow, Ireland) and the anti-rat IgG-FITC secondary antibody produced in rabbit (1:50, 1 h; Sigma-Aldrich, Darmstadt).

## Supplementary Information

**Additional file 1: Fig. S1.** Cell wall discs of *Actinotaenium* cf. *silvae*-*nigrae* excised by *Orciraptor agilis*. **Fig. S2.** SDS-PAGE of purified *Oa*GH5_5P protein produced in *Pichia pastoris*. Different volumes of sample (0.1–5 μl) were loaded to the wells. **Fig. S3.** Activity of *Oa*GH5_5E after pre-treatment at different temperatures reveals low temperature stability of the GH5_5 domain. **Fig. S4.** Western blot of whole cell lysate of *Orciraptor agilis* (increasing concentrations from left to right) treated with the anti-GH5_5 pAb.

**Additional file 2: Video S1.** Localisation of the GH5_5 endocellulase visualised by a 3D reconstruction from CLSM data. Three *Orciraptor* cells were fixed and immunostained during attack on *Actinotaenium* cf. *silvae-nigrae*.

**Additional file 3. **Codon-optimised sequence of the GH5_5 domain of *Orciraptor agilis* used for cloning.

## Data Availability

The datasets analysed during the current study are available from the corresponding author on reasonable request.

## Abbreviations

pAb: Polyclonal antibody
GH5_5: Glycoside hydrolase family 5, subfamily 5
PHA: Phalloidin
CFW: Calcofluor White
a.a.a.: After addition of algae
ORF: Open reading frame
Azo-CM-Cellulose: Chromogenic carboxymethyl cellulose
DNS: 3,5-Dinitrosalicylic acid
CMC-Na: Carboxymethyl cellulose sodium salt
HEC: Hydroxyethyl cellulose
IPTG: Isopropyl thio-β-D-galactoside
ELISA: Enzyme-linked immunosorbent assay
