# Supplementary material for: Experimental evidence for enzymatic cell wall dissolution in a microbial protoplast feeder (Orciraptor agilis, Viridiraptoridae)
Source: BMC Biol. 2022 Dec 5;20:267. doi: 10.1186/s12915-022-01478-x (PMC9721047; doi:10.1186/s12915-022-01478-x)
Supplement: Supplementary file 1 — Additional file 1: Fig. S1. Cell wall discs of Actinotaenium cf. silvae-nigrae excised by Orciraptor agilis. Fig. S2. SDS-PAGE of purified OaGH5_5P protein produced in Pichia pastoris. Different volumes of sample (0.1–5 μl) were loaded to the wells. Fig. S3. Activity of OaGH5_5E after pre-treatment at different temperatures reveals low temperature stability of the GH5_5 domain. Fig. S4. Western blot of whole cell lysate of Orciraptor agilis (increasing concentrations from left to right) treated with the anti-GH5_5 pAb. [file 12915_2022_1478_MOESM1_ESM.pdf]

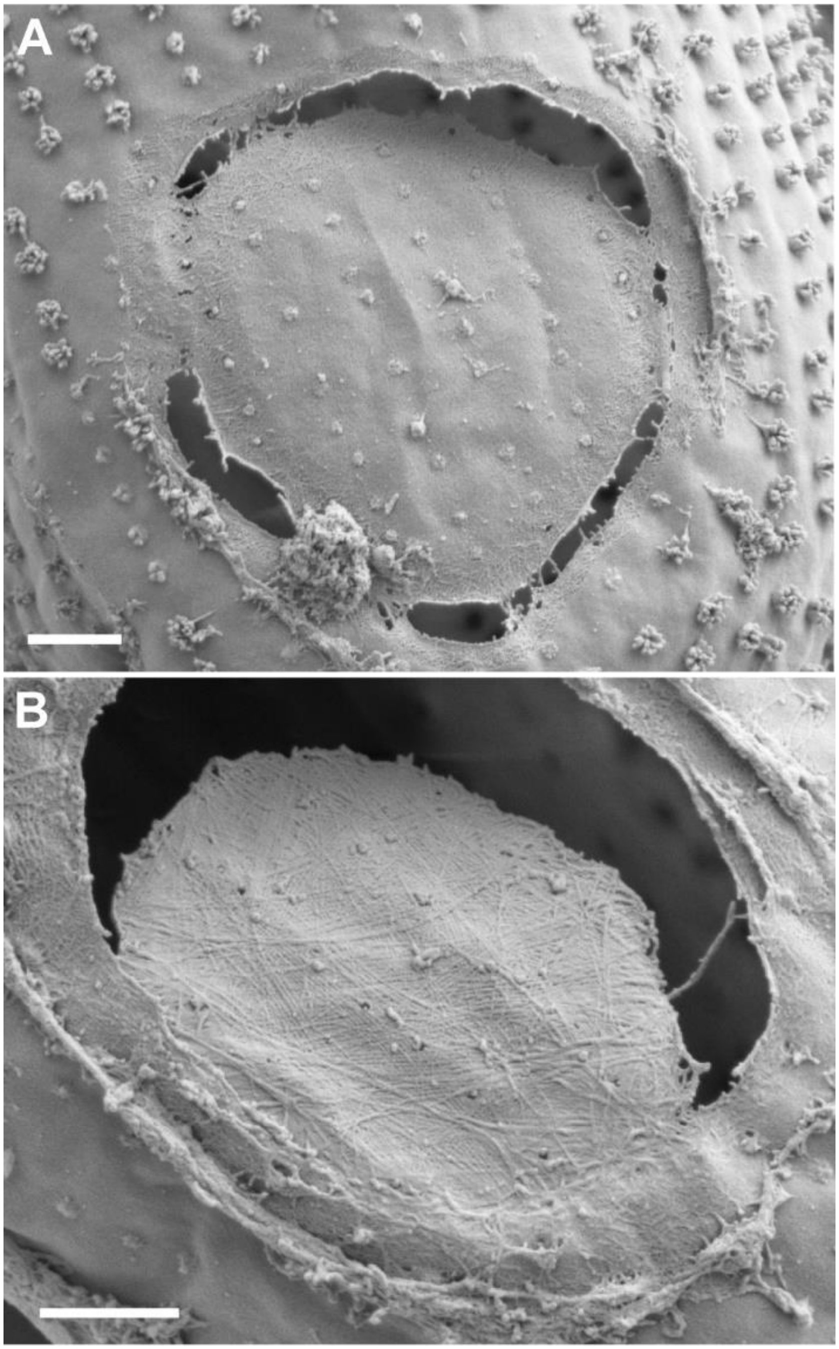

Fig. S1: Cell wall discs of *Actinotaenium* cf. *silvae-nigrae* excised by *Orciraptor agilis*. **A** Disc with a smooth surface (pectic substances) and partially degraded rosettes. **B** Disc with exposed cellulose microfibrils (pectic substances and rosettes on top are removed). Scale bars = 1 µm.

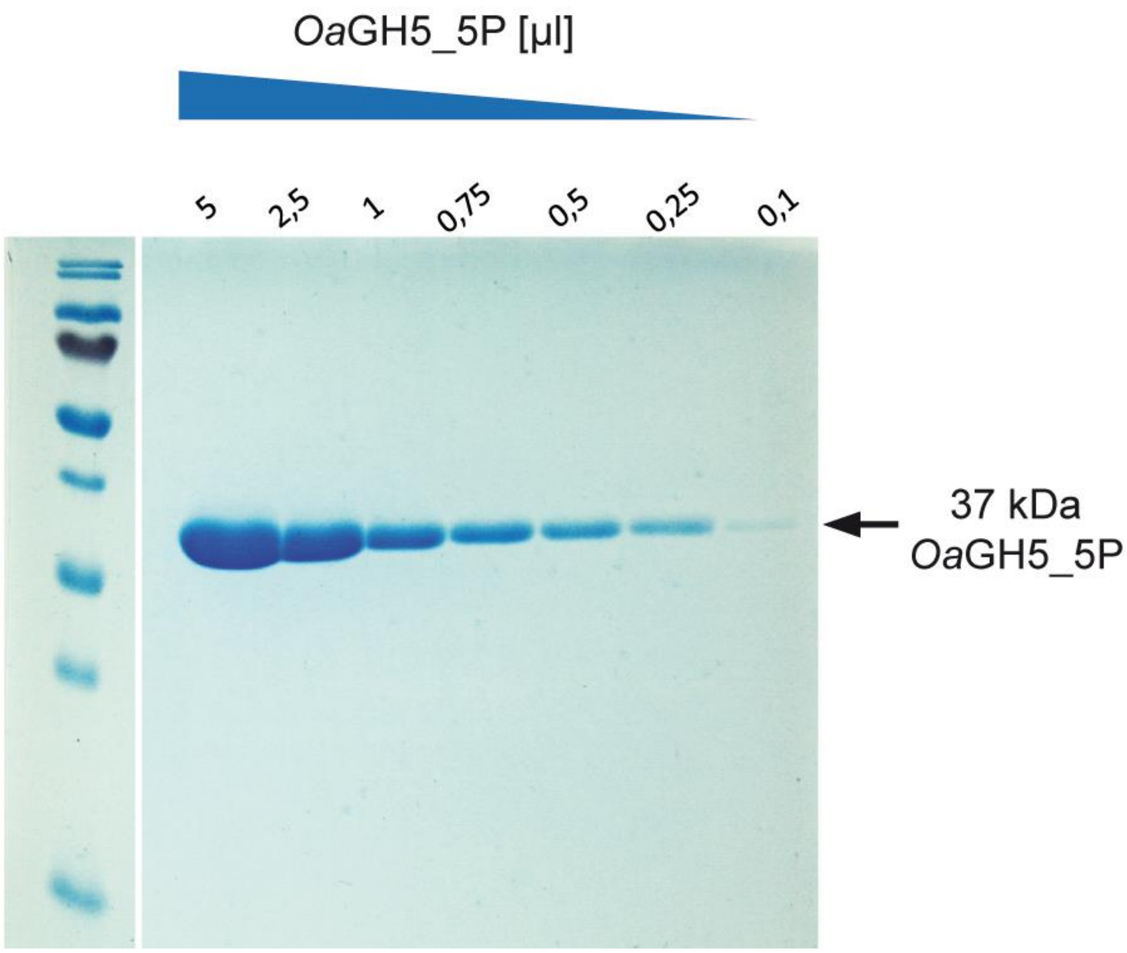

Fig. S2: SDS-PAGE of purified *Oa*GH5_5P protein produced in *Pichia pastoris*. Different volumes of sample (0.1–5 µl) were loaded to the wells.

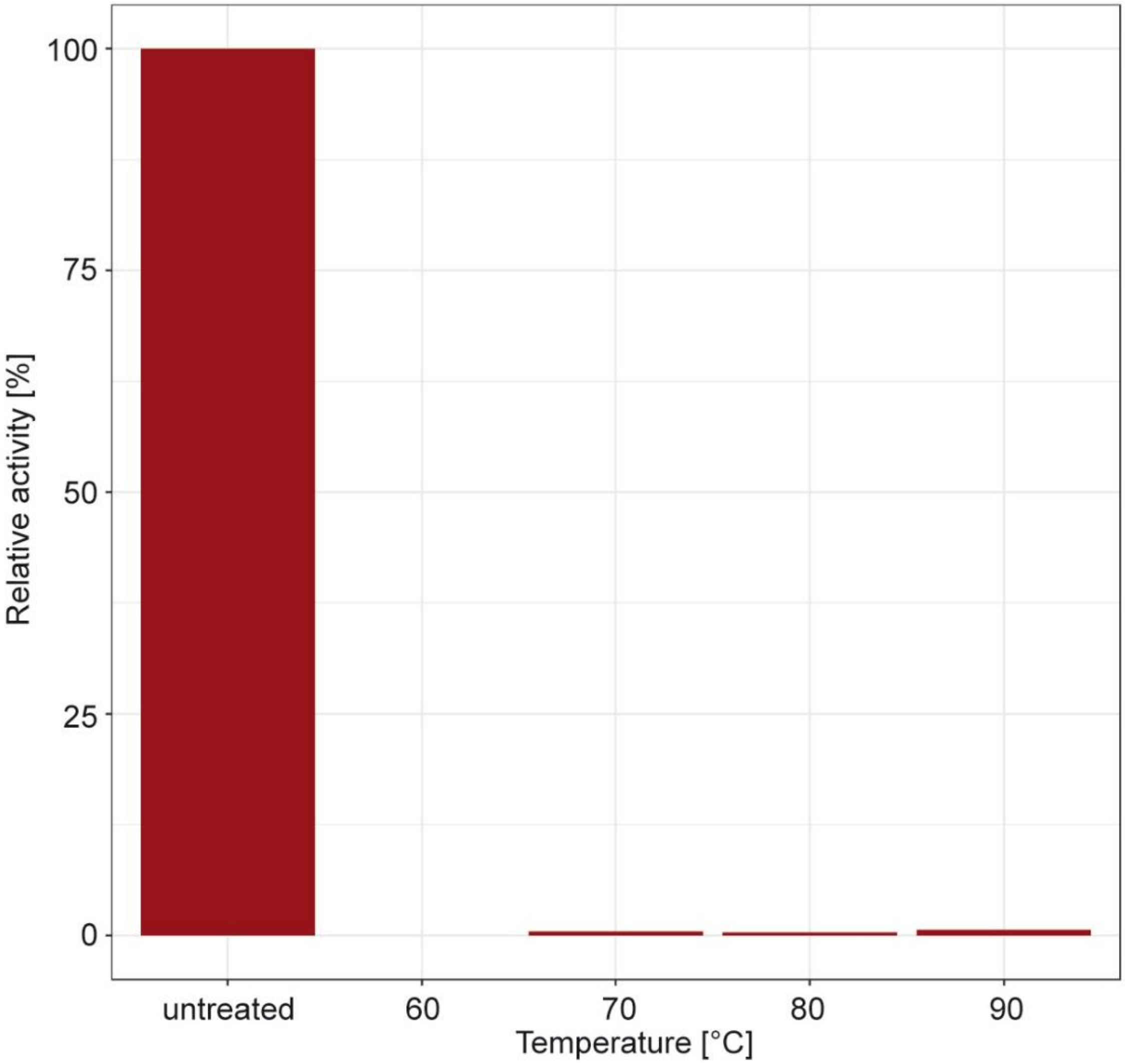

Fig. S3: Activity of *Oa*GH5_5E after pre-treatment at different temperatures reveals low temperature stability of the GH5_5 domain.

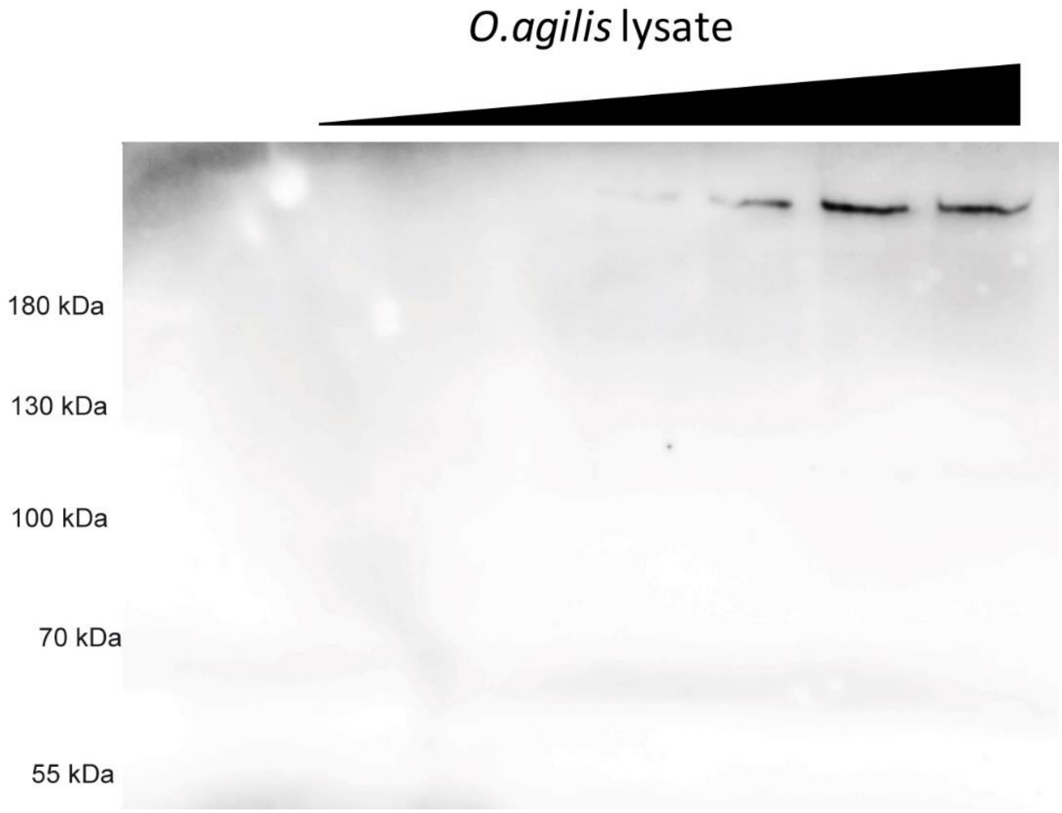

Fig. S4: Western blot of whole cell lysate of *Orciraptor agilis* (increasing concentrations from left to right) treated with the anti-GH5_5 pAb.
