# Supplementary material for: Experimental evidence for enzymatic cell wall dissolution in a microbial protoplast feeder (Orciraptor agilis, Viridiraptoridae)
Source: BMC Biol. 2022 Dec 5;20:267. doi: 10.1186/s12915-022-01478-x (PMC9721047; doi:10.1186/s12915-022-01478-x)
Supplement: Supplementary file 3 — Additional file 3. Codon-optimised sequence of the GH5_5 domain of Orciraptor agilis used for cloning. [file 12915_2022_1478_MOESM3_ESM.pdf]

Additional file 3

>*Oa*GH5_5_codon-optimised

ATGGGCGCGCACCTGAAGTACACCGGTGTTACCGAGAGCGGTCTGGAGTTCGGTTATAGCGTTGCGGGCGTG
GGTAGCGGTCCGGGTGTTTGCCAGCCGACCGCGGCGAGCTACAGCTTCATGGTGGCGAACAAGCTGAACCAC
GTGCGTGTTGCGTTTCGTTGGGAGCGTATCCAGCCGCAACTGAACGGTCCGCTGGACCCGACCGAACTGGGTA
AAGTTACCAGCGCTGTGAGCACCGCGTTTGCGAGCGGTCTGCAGTATGTGCTGGTTGACGTGCACAACTACGC
GGATTATGCGGGCACCCCGATTGGTCAGGGTGCGGTTACCATTGCGGCGTTCGCGAACCTGTGGAGCCGTCTG
GCGGTGGTTTTTACCCAGAGCAACATCGTTCTGGGTCTGATGAACGAACCGGTGGGTCCGCAGAGCGCGGGT
CAGGGTGGTGGCACCATGACCACCGAAACCTGGCTGGCGGCGGTGAACGCGGCGATTGCGGCGATTCGTGC
GCAAGGCAACACCAACCTGATTACCGTTCCGGGTCTGGGTTACACCGGCGCGCACGTGTGGGCGAGCAACCC
GGCGTACTATGGCACCAGCAACAGCCTGGTTATGCAGAACGTGGTTGACCCGCTGAACAACTACGTGTATGAG
ATCCACCAATATCTGGATAGCAGCACCGGTTTTGCGGGCACCGCGACCGACTGCGTTGATGGCCCGACCATCA
TTAGCCAGTTTACCGGTGTGATTCAATGGGCGACCATGTTCAACAAGAAACTGTGGCTGGGCGAGTTTGCGGC
GGCGAACAGCCCGGTTTGCCAGAGCAGCATCACCGCGCTGCTGAACTTCCTGGAAGCGAACAGCAACATTTTT
GTTGGTTGGACCTGGTGGAGCGCGGGTCCGTGCTGGGGTAACTACATGTTTAGCCTGGAACCGGGCACCGCG
AACCCGCAAATCAACTGGATTAGCCCGTTTGGCGCGCTGAACACCGGTGCGACCAGCGCGCCGACCACCCTG
